# Effect of the antifungal drug ciclopirox on the inhibition of HMGA2-mediated oncogenic capacity in ACHN renal cell carcinoma

**DOI:** 10.3389/fphar.2026.1723954

**Published:** 2026-02-13

**Authors:** Xujie Liu, Jiahao Su, Lingling Song, Qizhong Fu, Xuan Li, Junying Li, Ying Liu, Jingyi Liao

**Affiliations:** 1 Department of Urology Surgery, Affiliated Zhongshan Hospital of Dalian University, Dalian, China; 2 College of Basic Medical Sciences, Dalian Medical University, Dalian, China; 3 Department of Nutrition and Food Hygiene, School of Public Health, Peking University, Beijing, China

**Keywords:** CPX, epithelial-mesenchymal transition, gene signature, high mobility group protein A2, renal carcinoma

## Abstract

Ciclopirox olamine (CPX), an off-patent antifungal agent with a broad antimicrobial spectrum, is used to treat fungal infections. In addition to its antifungal effects, it inhibits tumor growth. However, little is known about the direct target proteins and anticancer mechanisms of CPX. The non-histone chromatin protein encoded by HMGA2, known as human high-mobility group A2, plays a crucial role in various biological processes such as cell cycle regulation, apoptosis induction, DNA damage repair, and the process of epithelial-mesenchymal transition. Increased HMGA2 expression is closely linked to tumor advancement, unfavorable prognosis, and inadequate response to therapeutic interventions. We found that CPX inhibited the level of the transcriptional regulator E2F1 in renal cancer cells by downregulating the expression of HMGA2, which led to a decrease in the expression of cell cycle protein D1 (CyclinD1) and cell cycle-dependent kinase 6 (CDK6), causing cell cycle disorders in renal cancer cells. Additionally, CPX significantly inhibited the proliferation, migration, and invasion of renal cancer cells *in vitro*. We also found that CPX exerted anti-tumor effects by inhibiting renal cancer cell proliferation and epithelial-mesenchymal transition (EMT) in in vivo xenograft mouse experiments. The TGF-β/Smads signaling pathway is linked to this phenomenon. These results provide robust evidence highlighting the potential for repurposing CPX in cancer treatment.

## 1 Introduction

Renal cell carcinoma (RCC) is the most common malignant tumor of the urinary tract, and its incidence has increased over the past few decades (Bray et al., 2018; Marques-Magalhães et al., 2021; Lorente et al., 2017; Lyskjær et al., 2024). In recent years, an increasing understanding of the molecular biology of RCC tumors has led to new therapeutic options (Wan et al., 2024; Gao et al., 2022), including targeted agents such as sorafenib, sunitinib, and temsirolimus. However, the prognosis of advanced renal cancers remains poor, suggesting that there is an urgent need for research into new therapeutic approaches (Liu et al., 2023; Barth et al., 2020; Ellinger et al., 2016; Garcia-Donas et al., 2015; Tippu et al., 2021). Recent studies have provided insights into the genetic and molecular alterations associated with RCC, such as the roles of VHL, PBRM1, SETD2, and BAP1 mutations in tumorigenesis and progression (Wan et al., 2024; Gao et al., 2022). These discoveries have paved the way for the development of novel targeted therapies and immunotherapies, which are showing promising results in clinical trials.

Ciclopirox olamine (CPX) is a synthetic hydroxypyridine analog with broad-spectrum antifungal activity that has been clinically used for the treatment of topical skin and nail fungal infections, CPX has been identified as a potential anti-tumor drug in recent years. Several studies have shown that CPX inhibits the proliferation of breast cancer, colorectal cancer, neuroblastoma, rhabdomyosarcoma, esophageal squamous carcinoma, human glioma, and other tumors (Huang et al., 2019; Zhou et al., 2010; Zhou et al., 2016; Zhou et al., 2019; Luo et al., 2011; Shen et al., 2017; Shen et al., 2018; Minde et al., 2014; Weir et al., 2011; Lu et al., 2022; Pádua et al., 2023; Yin et al., 2022; Chen et al., 2022; Qi et al., 2020; Su et al., 2021; Radadiya et al., 2021). However, to our knowledge,the effects of CPX on renal cancer have yet to be reported. The mechanism of CPX’s anti-cancer effects involves the disruption of intracellular iron homeostasis and inhibition of ribonucleotide reductase, which leads to DNA damage and cell cycle arrest. Given these findings, it is critical to explore CPX’s potential therapeutic effects on RCC and understand the underlying mechanisms involved.

HMGA2, which is in charge of encoding a non-histone protein in chromatin, plays a crucial role in several biological functions such as cell cycle regulation, apoptosis, repair of DNA damage, and the transition from epithelial to mesenchymal state. Studies indicate that an upregulation of HMGA2 is linked to the advancement of tumors, unfavorable prognosis, and inadequate response to treatment (Xia et al., 2015; Kou et al., 2018; Mansoori et al., 2020; Zhang et al., 2019; Cheraghi-Shavi et al., 2023; Pallante et al., 2015; Wu and Wei, 2013; Unachukwu et al., 2020; Qin et al., 2014; Liu et al., 2014; Langelotz et al., 2003). Our previous study showed that downregulation of HMGA2 expression could significantly inhibit the proliferation and invasive ability of renal cancer cells, promote apoptosis, and inhibit the development and progression of renal cancer. This suggests that HMGA2 expression is closely related to tumor formation, progression, and metastasis, and is expected to be an important target for renal cancer treatment (Liu et al., 2022; Liu et al., 2017; Zhu X. et al., 2023).

In our previous investigation, we observed that the expression of HMGA2 mRNA and protein was greater in the human kidney cancer cell line ACHN than in the human kidney cancer cell lines 786-O and 769-P.

In this study, we provided evidence showing that CPX effectively impedes the growth of ACHN renal cancer cells. Furthermore, our findings indicate that CPX induces cell cycle arrest specifically at the G1 phase. Additionally, our *in vivo* experiments demonstrate that CPX not only inhibits tumor growth but also effectively suppresses metastasis. Further mechanistic studies suggested that CPX can inhibit the expression of HMGA2, target E2F1/Cyclin D1/CDK6 and that disruption may contribute to cell cycle progression in renal cancer, providing a new tool for the treatment of renal cancer with good prospects for clinical application.

## 2 Materials and methods

### 2.1 Cell culture and chemical

ACHN cells were obtained from Jiangsu KGI Biotech Co. and cultured in 90% MEM medium (KeyGen Biotech Co., Ltd. KGM41500-500) + 10% FBS in an incubator at 37 °C, 5% CO_2_, and saturated humidity. Ciclopirox (CPX) and Sorafenib were purchased from TargetMol. Based on the information, we set the concentration of sorafenib in our cell experiments to 5 μM (Lee et al., 2022; Tei et al., 2015; Takeuchi et al., 2010; Kususda et al., 2012). Antibodies against HMGA2 (ER62068), E2F1 (ET1701-73), CyclinD1 (ET1601-31), E-cadherin (ET1607-75), N-cadherin (ET1607-37), Smad2 (ET1604-22), Smad3 (ET1607-41), TGFR II (ER 1917-66), and Snai1 (ER1706-22) were purchased from HuaBio (Hangzhou, China), and CDK6 (ab124821) antibody was purchased from Abcam (Cambridge, United Kingdom). The whole Protein Extraction Kit, BCA Protein Content Assay Kit, SDS-PAGE Gel Preparation Kit, Pre-stained Protein Molecular Weight, 5×SDS-PAGE Protein Sampling Buffer, 1×Tris-Glycine Protein Electrophoresis Buffer, Western blotting Assay Kit, and Annexin-V FITC/PI Cell Cycle Detection Kit were obtained from KeyGen Biotech Co., Ltd. (Nanjing, China). The 6-well cell culture plates were purchased from Corning, Inc. (United States).

### 2.2 CCK8 cell proliferation assays

Cells were digested, counted, and prepared into a cell suspension at a concentration of 4 × 10^3^ cells/mL. Then, 100 μL of cell suspension was added to each well of a 96-well cell culture plate (United States Corning Incorporated 3,599). The 96-well cell culture plate was incubated at 37 °C and 5% CO_2_ for 24 h. To achieve the desired concentration, the drug was diluted with a complete medium. Then, 100 μL of the medium containing the corresponding drug was added to each well. Simultaneously, a negative control group was prepared. The 96-well cell culture plates were incubated at 37 °C and 5% CO_2_ for 72 h. The 96-well plates were stained with CCK-8 and the OD values were determined at 450 nm. Statistical Package for the Social Science (SPSS) version 24.0, was used to calculate the IC50 by means of odds-unit weighted regression.

### 2.3 Clone formation experiment

Monolayers of logarithmically growing cells were dispersed in single-cell suspensions and counted using a standard passaging method. The cell suspension was diluted in gradient multiplicity, and 500 cells were inoculated into 6-well cell culture plates (Corning Incorporated 3,516, United States) containing 2 mL of culture medium, and the plate was gently shaken in the direction of “ten” to make the cells evenly dispersed. The cell culture plate was transferred into a CO_2_ incubator at 37 °C, 5% CO_2_, and saturated humidity, and left for 24 h for the cell to attach to the wall. After 10 days, the culture solution was discarded, the culture was terminated, and the plate was washed twice with PBS by careful immersion. Five mL of anhydrous ethanol was added for 15 min to fix the cells. After discarding the fixative, Richard-Allan Scientific™ (ThermoFisher 3121TS, United States) solution was added for 10–30 min, and the staining solution was slowly rinsed with running water and air dried. The number of clones was counted and every 50 or more cell clusters was considered one clone.

### 2.4 Transwell assay for detection of cell invasion

Cells in the logarithmic growth phase were digested and inoculated into 6-well plates. On the following day, after the cells were attached to the wall, the cells were withdrawn from serum and starved using incomplete medium for 24 h. Matrigel matrix gel (BD 356234, United States) was placed at 4 °C overnight to melt. Dilute the diluted Matrigel 2x with incomplete culture medium, add 30 μL of the diluted Matrigel to the upper Transwell chamber, and incubate at 37 °C for 120 min to allow the Matrigel to polymerize into a gel. Harvest cells by digestion with 0.25% trypsin. The cell density was adjusted to 1 × 10^5^ cells/mL using incomplete medium. Then, we added 100 µL of cell suspension to a Transwell (Corning Incorporated 3,422, United States), and 500 µL of medium containing 20% FBS was added to the lower chamber. The 24-well cell culture plate (Corning Incorporated 3,514, United States) was incubated at 37 °C in a 5% CO_2_ incubator for 12 h. For cell counting, the cells in the matrix gel and upper chamber were wiped off with a cotton swab, removed from the Transwell, inverted, air-dried, and the 24-well plate was filled with 500 µL of medium containing 0.1% Crystal Violet (Sigma C3886, United States), and the small chamber was placed in it, so that the membrane was submerged in the dye.

### 2.5 Detection of cell migration by scratch method

Cells in the logarithmic growth phase were digested and inoculated into a 6-well cell culture plate (Corning Incorporated 3,516, United States). On the next day, when the cell aggregation reached approximately 80%, the cells were uniformly scribed in the 6-well plate with the tip of a sterile gun. The floating cells were washed with PBS, and the culture medium was replaced with fresh culture medium and cultured. After 24 h of incubation, cells were removed, photographed (×100 magnification), and the distance of cell migration was measured.

### 2.6 PI single staining method for cell cycle detection

The cells were washed twice with PBS (centrifugation 1,000 rpm for 5 min) to collect 5 × 10^5^ cells. The prepared single-cell suspension was fixed with 70% ethanol by volume for 2 h (or overnight) and stored at 4 °C. The fixative was washed with PBS before staining. Then, 100 μL of RNAse A was added and mixed with 400 μL of PI staining for 30 min at 4 °C in the dark. The red fluorescence was recorded at an excitation wavelength of 488 nm using the CytoFLEX flow cytometry system (Beckman Coulter, Brea, CA, United States).

### 2.7 Detection of apoptosis by Annexin-V FITC/PI double staining method

Cells in the logarithmic growth phase were digested and inoculated into 6-well plates, and the corresponding spiked culture solution was added according to the group setting for 72 h. Cells were collected by digestion with 0.25% trypsin without EDTA. Afterwards, cells were washed twice with PBS and centrifuged at 1,000 rpm for 5 min. Approximately 5 × 10^5^ cells were collected, and 500 μL of Binding Buffer was added to suspend the cells. Then, 5 μL of Annexin V-FITC was added to mix with the cells, followed by 5 μL of Propidium Iodide. After incubation at room temperature in the dark for 5–15 min, apoptosis was detected using flow cytometry (BECKMAN COULTER CytoFLEX).

### 2.8 Xenograft studies

A total of 16 BALB/c female nude mice were purchased from Shanghai Lingchang Biotechnology Co., Ltd. Co. Ltd. (Shanghai, China). All mice were maintained in standard laboratory cages (room temperature 20 °C–26 °C, relative humidity 40%–70%, and 12 h of alternating light and dark) with free access to food and water. All animal experimental protocols were approved by the Experimental Animal Ethics Committee of the Zhongshan Hospital, Dalian University.

The cultured human renal adenocarcinoma cells ACHN cell suspension was collected at a concentration of 5X10^7^ cells/ml and inoculated subcutaneously into the axilla of the right forelimb of the mice at 0.1 mL each. Mouse graft tumors were measured with Vernier calipers to determine the diameter of the graft tumor, and the animals were randomly divided into four groups of four animals each when the tumor grew to approximately 200 mm^3^: blank control group, low-dose CPX-treated group (10 mg/kg), high-dose CPX-treated group (30 mg/kg), and sorafenib as the positive control group. According to the relevant data, we set the concentration of sorafenib at 20 mg/kg in animal experiments (Tei et al., 2015; Takeuchi et al., 2010; Kususda et al., 2012). All mice were injected intraperitoneally for 16 days. The anti-tumor effects of the treatments were observed dynamically by measuring the tumor diameter. The tumor volume was calculated in mm^3^ as (width^2^ x length)/2. Mice were sacrificed by cervical dislocation at the end of the experiment, tumors were surgically removed, and body weight was measured. Death was verified by the cessation of respiratory movements. In order to adhere to the principle of humane endpoints, the following measures were taken during the experiment to minimize the use of animals while ensuring the scientific rigor and humanity of the experiment. First, adequate practice and prior knowledge of modeling techniques were conducted to significantly reduce the failure rate of animal modeling, thus ensuring the confidence and validity of the experimental results. Second, it was ensured that animals were anesthetized during all experimental manipulations to minimize potential pain and discomfort during the experimental process. In addition, experimental ethical norms were strictly adhered to and clear humanitarian endpoints were developed to suit the objectives of this study. The specific criteria were as follows: i) when the pain suffered by the animals was not caused by the experiment itself and was unexpected before the start of the experiment, e.g. animals with congenital defects; ii) if it was anticipated before the experiment that the animals might feel pain, but the pain was more than expected during the actual procedure, e.g. severe nasal and oral bleeding, severely abnormal behavior and other conditions; iii) if the animal’s pain is caused by the experiment itself and this pain is anticipated before the experiment begins. Once any of these criteria were adhered to during the course of the experiment, the animals were immediately euthanized to reduce their suffering. Once these criteria were met, all animals in this study were euthanized before the end of the experiment, ensuring that no animal would die for any unnecessary reason during the course of the experiment. Animal welfare was always adhered to, ensuring that the experiments ran smoothly while maintaining the accuracy and reliability of the results. A total of 16 mice were used and euthanized in the experiment, with no deaths occurring before the end of the experiment. Animal death was confirmed by the observation of respiratory and cardiac arrest and pupil dilation for at least 10 min. All animal welfare considerations were taken, including efforts to minimize suffering and distress, the use of analgesics or anesthetics, and special housing conditions. All experimental procedures and methods described in the study were approved by and carried out in accordance with the Ethics Committee of the Affiliated Zhongshan Hospital of Dalian University. We confirmed that the study was carried out in compliance with the ARRIVE guidelines for the reporting of animal experiments. Ethical IRB number: KYS20230195.

### 2.9 Western blotting analysis

Cell lysates were harvested from ACHN cells or xenograft tumors using RIPA cell lysis buffer according to the manufacturer’s instructions. Total protein concentration was determined by the biszirconate method (Bradford method, BCA). Protein samples were separated using sodium dodecyl sulfate polyacrylamide gel (SDS-PAGE) and transferred to PVDF membrane. Proteins were then immunoblotted with anti-HMGA2, anti-E2F1, anti-CyclinD1, anti-CDK6, anti-E-cadherin, anti-N-cadherin, anti-Smad2, anti-Smad3, anti-TGFRII, and anti-Snai1 antibodies. The membranes were then washed and developed using ECL reagent (KGP116 Nanjing, China), and images were captured and analyzed using a gel imaging system (Bio-Rad ChemiDoc MP Imaging System, United States).

### 2.10 Real-time polymerase chain reaction (RT-PCR) analysis

Total RNA was extracted using the TRIzol™ method according to the manufacturer’s instructions. A total of 5 μg of total RNA was reverse transcribed into cDNA using the cDNA First Strand Synthesis Kit (TaKaRa RR036B, Japan). Primer sequences are shown in Table 1. RT-PCR was performed using 2× real-time fluorescence quantitative PCR premix (SYBR Green) on an ABI StepOnePlus real-time fluorescence quantitative PCR system (Thermo Fisher Scientific, Waltham, MA, United States).

**TABLE 1:** RT-PCR primer sequences.

Gene	Forward primer	Reverse primer
CDK6	5'-GGTGACCAGCAGCGGACAAAT-3'	5'-TGTACCACAGCGTGACGACCA-3'
CyclinD1	5'-CGCCCTCGGTGTCCTACTTCAA-3'	5'-GTTCCTCGCAGACCTCCAGCAT-3'
E2F1	5'-GCCACTGACTCTGCCACCATAG-3'	5'-GCTCCAGGCTGAGTAGAGACTG-3'
GAPDH	5'-AGATCATCAGCAATGCCTCCT-3'	5'-TGAGTCCTTCCACGATACCAA-3'
HMGA2	5'-ACTTCAGCCCAGGGACAACCT-3'	5'-TTGGTTCTTGCTGCTGCTTCCT-3'
E-cadherin (CDH1)	5'-CTACAATGCCGCCATCGCTTAC-3'	5'-GGTGACCACACTGATGACTCCT-3'
N-cadherin (CDH2)	5'-CCTGAGGGATCAAAGCCTGGAA-3'	5'-TGGAGCCTGAGACACGATTCTG-3'
SMAD2	5'-CCATTCACGCCGCCAGTTGT-3'	5'-TTCCTGCCCATTCTGCTCTCCT-3'
SMAD3	5'-CCTCGCAGCCATCCATGACTGT-3'	5'-ACGCCTCTTCCGATGTGTCTCC-3'
Snai1	5'-CCTCGCTGCCAATGCTCATCT-3'	5'-GCCTTTCCCACTGTCCTCATCT-3'
TGFBR2	5'-ACGACCTAACCTGCTGCCTG-3'	5'-AGTTCCCACCTGCCCACTGTT-3'

### 2.11 Hematoxylin & Eosin (HE) staining

Tissue dewaxing was performed as usual, hydration was performed, and the sections were soaked in xylene for 5 min, replaced with xylene, soaked for another 5 min, and rehydrated in graded ethanol (100, 95, 85, and 70%). The sections were soaked for 5 min in 70% ethanol and washed in PBS for 3 min thrice. Finally, the sections were stained with HE.

### 2.12 Statistical analysis

Statistical analyses were performed using the SPSS software (version 25.0; SPSS Inc., Chicago, IL, United States). Data are expressed as mean ± standard deviation. Three or more groups were compared by one-way analysis of variance followed by Bonferroni or Tukey’s *post hoc* multiple comparisons test. P ≤ 0.05 was considered to indicate a statistically significant difference.

## 3 Results

### 3.1 CPX inhibits the growth and proliferation of human renal cancer ACHN cells

**FIGURE 1:**
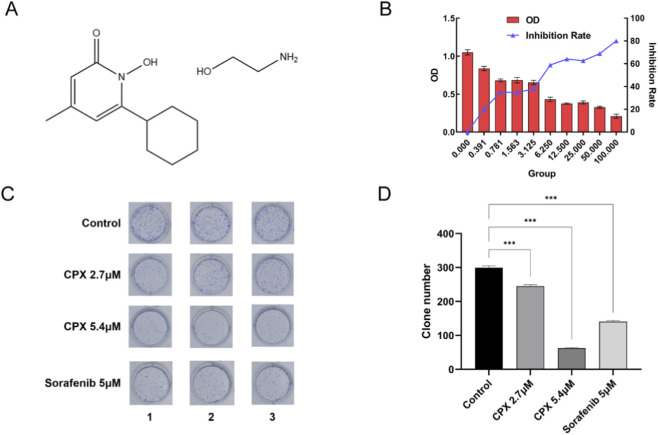
Antitumor effects of CPX. **(A)** Chemical formula of CPX. **(B)** OD values and inhibition rates of kidney cancer ACHN cells after 72 h of treatment with different concentrations of CPX. **(C)** Clone formation identification. **(D)** Clone number treatment. *P < 0.05, **P < 0.01; ***P < 0.001.

Ciclopir oxamine (CPX), an organic compound with the chemical formula 14H24N2O3 (Figure 1A), is a class of broad-spectrum antifungal agents, which has attracted extensive attention from tumor therapeutics researchers in recent years as it has been determined to have anti-tumor effects. To investigate whether CPX could effectively inhibit renal cancer ACHN cells, we examined the cell inhibition rate at different concentrations of CPX. As shown in Figure 1B, the results of the CCK8 assay illustrated that the OD value of CPX on ACHN cells gradually decreased, and the inhibition rate increased with an increase in CPX concentration after 72 h (P < 0.001). The IC50 = 5.407 μM was calculated through the chance unit-weighted regression method, suggesting that CPX can produce toxic effects on tumor cells at lower concentrations, and the higher the concentration of the drug, the greater the toxic effect on ACHN cells. Based on the results of the preliminary experiments, we set the CPX concentrations to 2.7 μM and 5.4 μM in subsequent experiments. As shown in Figure 1C, in the cloning experiments, the CPX and sorafenib positive control group significantly inhibited the proliferation of ACHN cells compared with the saline control group, and the inhibitory effect of CPX was stronger at high concentrations of CPX (Figure 1D).

### 3.2 CPX inhibits migration and invasion of human renal cancer ACHN cells

**FIGURE 2:**
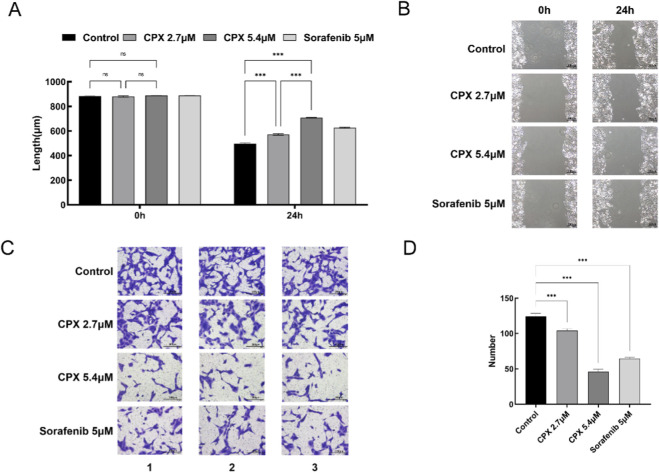
Migration and invasion of human renal cancer ACHN cells. **(A,B)** Effect of CPX on ACHN cell migration in renal carcinoma cells tested by the scratch wound healing assay. **(C,D)** The effect of CPX on migration and invasion of renal cancer ACHN cells was further determined using a Transwell assay. *P < 0.05, * *P < 0.01, * **P < 0.001.

In the cell scratch assay, the migration distance of cells was measured after 24 h, as shown in Figures 2A,B. Compared with that in the blank control group, the migration ability of the CPX and sorafenib groups was significantly weaker, and the inhibitory effect of the high concentration of CPX was more pronounced (control: 496.73 ± 5.23 μm; 2.7 μM CPX: 572.02 ± 5.94 μm; 5.4 μM CPX:709.18 ± 3.49 μm; 5 μM Sorafenib:626.2 ± 3.71 μm,P < 0.001).

In the Transwell assay, the number of membrane-penetrating ACHN cells after 12 h of treatment with different drug concentrations is shown in Figures 2C,D. Compared with the blank control group, the CPX and sorafenib positive control groups had stronger inhibitory effects on the invasive ability of renal cancer cells, and the penetration ability of ACHN cells was weakened. The inhibitory effect of a high concentration of CPX was evident.

### 3.3 CPX blocks the renal cancer ACHN cell cycle by inhibits G1/S transition

**FIGURE 3:**
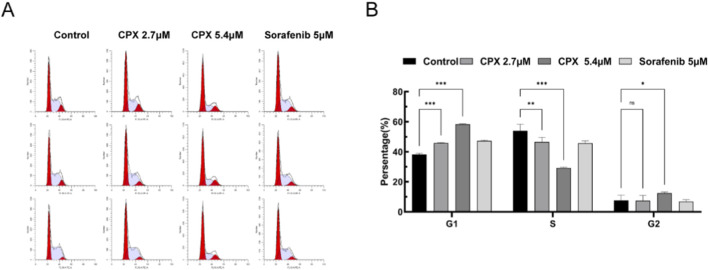
Renal cancer ACHN cell cycle analysis. **(A)** Flow cytometry analysis of CPX- and Sorafenib-treated renal cancer ACHN cells. **(B)** Bar graph showing the percentage of cells in each cell cycle phase. *P < 0.05, **P < 0.01, * **P < 0.001.

Based on the specific binding of propidium iodide and other fluorescent dyes to DNA, the effect of CPX on the cycle of ACHN renal cell carcinoma cells was analyzed by flow cytometry. The proportions of ACHN cells in the G0/G1, S, and G2/M phases in each group are shown in Figure 3A. Cell cycle progression was determined using flow cytometry, as shown in Figure 3B. Compared to the blank control group, the proportion of cells in the G0/G1 phase was significantly higher in the CPX and sorafenib groups after treatment (P < 0.001).

### 3.4 CPX induces apoptosis in renal cancer ACHN cells in an *in vitro* assay

**FIGURE 4:**
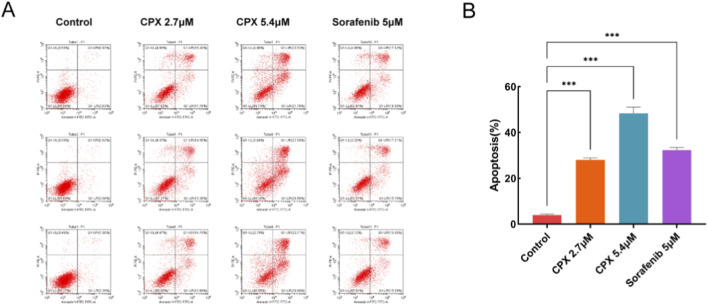
Apoptosis in renal cancer ACHN cells. **(A)** Flow cytometry and **(B)** apoptotic effect analyses of renal cancer ACHN cells treated with CPX or Sorafenib for 72 h. *P < 0.05, **P < 0.01, ***P < 0.001.

Previous studies have shown that ACHN cells treated with HMGA2-siRNA interference achieve gene silencing and induce apoptosis. To verify whether CPX could produce apoptotic effects on renal cancer ACHN cells, renal cancer cells were cultured for 72 h with each reagent concentration and then detected using flow cytometry. The results show that, compared with that of the blank control group, the difference in the apoptosis rate of CPX-treated and the positive control groups was significantly higher, with a concentration-dependent increasing trend (P < 0.001) (Figures 4A,B).

### 3.5 Inhibition of mouse xenograft tumor growth by CPX administration

**FIGURE 5:**
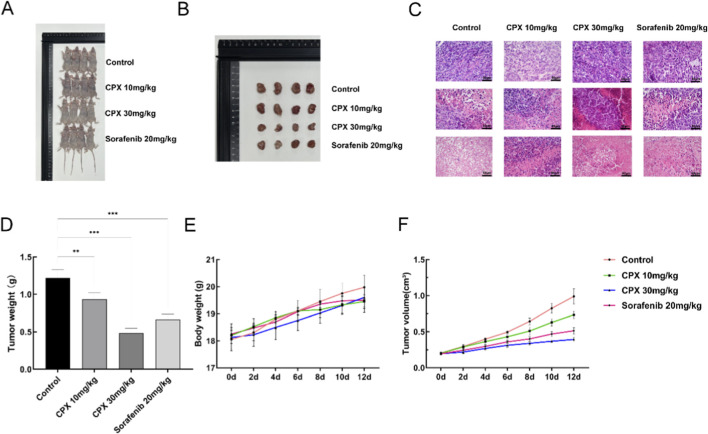
Analysis of mouse xenograft tumor growth. **(A)** Images of nude mice in treatment and control groups. **(B)** Tumor images of nude mice in treatment and control groups. **(C)** H&E staining of tumor sections. **(D)** Tumor weights of nude mice in treatment and control groups. **(E)** Tumor volume in nude mice in treatment and control groups. Volume: V=(width^2^ × length)/2. n = 8. **(F)** Body weights of nude mice in treatment and control groups. *P < 0.05, **P < 0.01, ***P < 0.001.

Histopathological analyses of mouse tumors were performed (Figures 5A–C), and H&E staining showed that the control tumor cells were dense and homogeneous with intensely stained nuclei, whereas nuclear sepsis, fragmentation and zones of broadly lysed necrosis were observed in the CPX-treated group. A renal cancer ACHN xenograft model was used to evaluate the inhibitory effects of CPX *in vivo* in order to further examine the anti-tumor potential of the compound. The animals were separated into two groups at random after the tumor volume reached about 200 mm^3^, a blank group, and the other group. Low-concentration CPX treatment group (10 mg/kg), high-concentration CPX treatment group (30 mg/kg), and sorafenib-positive control group (20 mg/kg), with four animals in each group. Tumor volume and body weight were recorded for statistical analysis. Compared to that in the control group, the tumor volume was significantly smaller (Figure 5D) and the tumor weight was also smaller than the control group (Figure 5E). This indicated that CPX significantly inhibited the growth of renal cancer ACHN cells *in vivo* (P < 0.001). Notably, there was no significant difference in mean weight between the control and treatment groups (Figure 5F).

### 3.6 CPX reduces the epithelial-mesenchymal transition (EMT) in renal cancer ACHN cells by inhibiting the TGF-β/Smads signaling pathway through down-regulation of HMGA2 protein expression level

**FIGURE 6:**
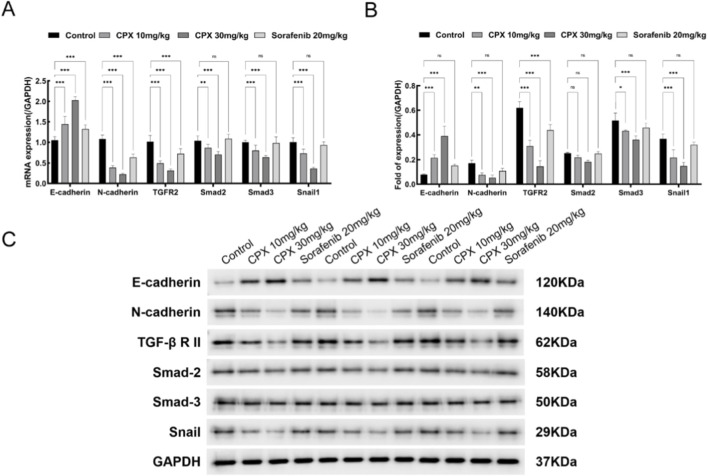
Effect of CPX on the expression of E-cadherin, N-cadherin, TGF-β R II, Smad-2, Smad-3, and Snail in tumor tissues. **(A)** Expression levels of E-cadherin, N-cadherin, TGF-β R II, Smad-2, Smad-3, and Snail in tumor tissues detected by qRT-PCR. **(B)** The expression levels of HMGA2, E2F1, Cyclin D1 and CDK6 in tumor tissues were examined by protein blotting **(C)**. Quantitation of the result of Western blot. Values are shown as mean ± SD. * P < 0.05 vs. control group, * ** P < 0.001 vs. control group.

EMT is crucial in cancer progression, and in the microenvironment of epithelial tissues various extracellular factors can induce EMT, one of which is TGF-β. TGF-β can significantly induce EMT through a set of specific mediator proteins and transcription factors, which constitute the framework of the TGF-β signaling pathway. Our previous studies have shown that the mechanism by which HMGA2 exerts its biological function is related to the TGF-β signaling pathway, in which HMGA2 can bind to Smad2 and Smad3 and form a complex with each other, inducing the expression of the target gene Snail, promoting the production of Snail protein, and triggering the occurrence of EMT in renal cancer cells, which promotes renal cancer cell occurrence, development and metastasis. In order to investigate the effects of CPX on the molecules related to TGF-β/Smads signaling pathway, we detected transforming growth factor-β receptor II (TGF-β R II), receptor-activated Smads family proteins (Smad-2, Smad-3), transcription factor Snail, and EMT-related proteins E-cadherin and N-cadherin. The qRT-PCR results showed that compared with the blank control group, the mRNA expression of N-cadherin, TGF-β R II, Smad-2, Smad-3, and Snail in the CPX group was significantly reduced, and the expression of E-cadherin was significantly increased (P < 0.001) (Figure 6A). In the Western blot experiment, compared with that in the blank control group, the protein expression of N-cadherin, TGF-β R II, Smad-2, Smad-3, and Snail in the CPX group was significantly reduced, and the expression of E-cadherin was significantly increased (P < 0.001) (Figures 6B,C). The results indicated that CPX inhibited the production of Snail, a key transcription factor for EMT, by inhibiting the expression of HMGA2, resulting in a decrease in the expression of N-cadherin, TGF-β R II, Smad-2, Smad-3, and Snail, and an increase in the expression of E-cadherin, thus inhibiting EMT in renal cancer cells by inhibiting HMGA2 expression.

### 3.7 CPX inhibits the E2F1/Cyclin D1/CDK6 signaling pathway by down-regulating HMGA2 protein levels, leading to cell cycle disruption in renal carcinoma ACHN

**FIGURE 7:**
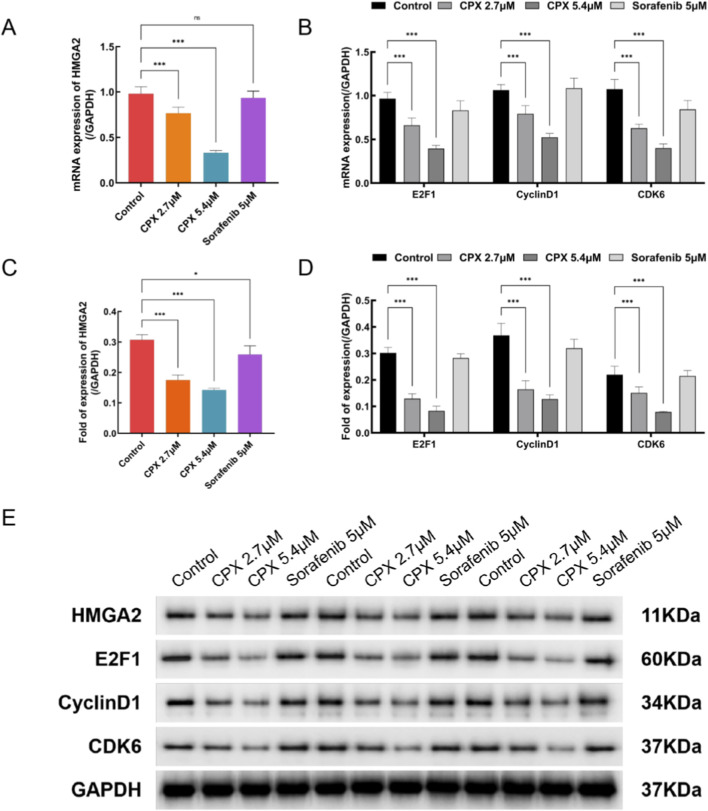
Effect of CPX on the expression of HMGA2,E2F1, Cyclin D1, and CDK6 in renal cancer ACHN cells. **(A)** Expression levels of HMGA2,E2F1, Cyclin D1 and CDK6 in tumor tissues detected by qRT-PCR. **(B)** The expression levels of HMGA2, E2F1, Cyclin D1, and CDK6 in renal cancer ACHN cells examined by protein blotting. **(C)** Quantitation of the result of Western blot. Values are shown as mean ± SD. * P < 0.05 vs. control group, * ** P < 0.001 vs. control group. **(D)** The expression levels of HMGA2, E2F1, Cyclin D1, and CDK6 in renal cancer ACHN cells examined by qRT-PCR. **(E)** Quantitation of the result of Western blot.

Previous studies have shown that HMGA2 plays an oncogenic role in renal cancer; is closely related to tumor formation, progression, and metastasis; and is an important target for renal cancer therapy. In this study, we examined the effect of CPX on HMGA2 expression in kidney cancer ACHN cells using RT-PCR and protein blotting. Compared with the control group, the expression of HMGA2 mRNA and protein in the CPX group was significantly inhibited, and the inhibitory effect was stronger in the high-concentration group (Figures 7A–C). These results indicate that CPX acts as an HMGA2 inhibitor in renal cancer cells.

Numerous lines of research have revealed that HMGA2 encourages tumor cell proliferation by upregulating the expression of cell cycle-related genes. Cell cycle disruption is thought to be a typical aspect of carcinogenesis. Specific reasons include the following: (1) HMGA2 induces an increase in the activity of the transcription factor E2F1 and promotes aberrant cell proliferation, thereby facilitating the transition to the G2/M phase of the cell cycle and inducing tumor growth. (2) HMGA2 increases the expression of cell cycle protein D1 (CyclinD1) and cell cycle-dependent kinase 6 (CDK6), promotes the formation of the CyclinD1 CDK6 complex, accelerates the G1 phase of the cell cycle, promotes the transformation of the G2/M phase, interferes with the cell cycle, and promotes tumorigenesis. (3) HMGA2 overexpression disrupts the DNA repair system. The mRNA expression of each group was detected through qRT-PCR, and the results showed that the protein expression of HMGA2 and the E2F1/Cyclin D1/CDK6 pathway was downregulated in the CPX-treated and positive control groups compared with that in the blank control group (Figure 7A). In Western blot experiments, the CyclinD1/CDK6/E2F1 pathway protein expression was significantly reduced in the CPX group compared with the blank control group, and the difference was statistically significant (P < 0.001) (Figures 7B,C). This indicates that CPX inhibited the expression of HMGA2 and suppressed the level of E2F1 in renal carcinoma ACHN cells, which resulted in decreased expression of CyclinD1 and CDK 6, leading to renal cancer cell cycle disorders.

## 4 Discussion

Kidney carcinoma is a typical malignant tumor of the urinary system. Despite considerable advancements in kidney cancer treatment, surveys have indicated that the prevalence of kidney cancer has increased greatly over the past 10 years, and the prognosis for patients is still not positive (Tippu et al., 2021). Approximately 45% of renal cancer patients have metastasis at the initial diagnosis, and 50% of renal cancer patients have tumor recurrence after surgery. This is mostly owing to the highly varied biological activity of kidney cancer, which makes it difficult to forecast using typical histological staging and grading (Lyskjær et al., 2024; Rydzanicz et al., 2013). Current treatments for renal cancer are mainly based on early surgical resection and late targeted drugs. Given the renal cancer cellular microenvironment and glycolipid metabolism, renal cancer is insensitive to radiotherapy, which increases the postoperative metastasis and recurrence rates of patients with renal cancer. Compared to other malignant tumors, renal cancer does not have specific tumor markers. Anti-tumor drugs commonly used in clinical practice have broad-spectrum anti-tumor activity with poor specificity and selectivity. Currently, 15 targeted and immunological drugs, mainly anti-angiogenic tyrosine kinase inhibitors (TKIs) and mTOR inhibitors, have been approved by the FDA for use in renal cancer. According to the results of a global multi-center clinical study, 30% of metastatic kidney cancer patients have primary resistance to molecularly targeted drugs, and those patients who are initially sensitive to the treatment will develop “secondary resistance” within about 1 year after receiving the treatment, which greatly affects the efficacy of molecularly targeted drugs (Rydzanicz et al., 2013; Takahashi et al., 2001; Kroeze et al., 2010; Leung and Kim, 2017). Therefore, it is vital to find novel therapeutic modalities to extend life and raise the survival rate of kidney cancer patients.

Ciclopirox is mainly used clinically to treat fungal infectious diseases and has great potential for the prevention and treatment of diabetes mellitus, Acquired Immune Deficiency Syndrome (AIDS), and cardiovascular diseases (Zhou et al., 2023). Several *in vitro* tests have shown that CPX can inhibit the proliferation of tumor cells, such as rhabdomyosarcoma, colon adenocarcinoma, esophageal squamous carcinoma, and human glioma, and can inhibit the generation of blood and lymphatic vessels (Zhou et al., 2016; Zhou et al., 2019; Shen et al., 2017; Lu et al., 2022; Zhou et al., 2023; Clement et al., 2002; Ihn et al., 2021; Zhu Y. et al., 2023; Feng et al., 2020; Kremer et al., 2019; Linden et al., 2003; Al-Dali et al., 2018). Tumor development and metastasis are inextricably linked to the generation of blood and lymphatic vessels. Pharmacological and toxicological profiles indicate that CPX is a relatively effective and safe antifungal drug. Systemic application in nude mice at a dose of 20–25 mg/kg/day can effectively inhibit tumor growth without showing significant toxicity (Huang et al., 2019; Zhou et al., 2010). CPX has multiple molecular targets, such as RR, DOHH/eIF5A, Wnt/β-catenin, VEGFR-3/ERK1/2, CDKs, members of the Bcl-2 family, and mTOR ((Zhou et al., 2010), (Eberhard et al., 2009; Kim et al., 2011; Sen et al., 2013)). CPX can induce apoptosis and anti-tumor metastasis and inhibit the proliferation of tumor cells through the above molecular targets, thus exerting a killing effect on tumor cells. The effects of CPX on these targets are primarily related to iron chelation (Niewerth et al., 2003). Tumor cells inevitably consume more iron to maintain a much higher proliferation rate than normal cells, and iron chelation usually has a greater effect on tumor cells.

There is a correlation between HMGA2 expression and renal cancer TNM stage and lymph node metastasis; the higher the tumor stage, the higher the expression of HMGA2, and the shorter the patient survival time. Recent studies have demonstrated that HMGA2 mRNA and protein expression in renal cancer tissues is higher than that in benign renal tumor tissues and normal renal tissues. (Zhang et al., 2019; Unachukwu et al., 2020; Liu et al., 2022; Ekanem et al., 2020; Xu et al., 2023; Wang et al., 2023; Cai et al., 2016). In the present study, we showed for the first time the inhibitory effect of CPX on the HMGA2-mediated oncogenic ability of renal cancer cells; CPX suppressed the expression of HMGA2 in renal cancer cells, which in turn caused cell cycle arrest and apoptosis and inhibited the proliferation, invasion, and migration ability of renal cancer cells. We discovered that the TGF-β/Smads signaling pathway is connected to the mechanism by which CPX affects kidney cancer cells. Through several particular transcription factors and mediator proteins, TGF- may greatly promote EMT. These specific mediator proteins, transcription factors, and TGF-β constitute the framework of the TGF-β signaling pathway, wherein TGF-β acts as a ligand, binds to the type II receptor (TGF-β R II), recruits and phosphorylates receptor-regulated Smads proteins (Smad-2, Smad-3), and after activation by phosphorylation of Smad-2, Smad-3, binds to cofactor (coSMAD) and forms a complex which accumulates as a transcription factor in the nucleus. It encourages the creation of Snail protein and the target gene’s expression, which both encourage EMT in renal carcinoma cells. In renal cancer cells, CPX was able to increase E-cadherin expression, decrease N-cadherin and Snail expression, and prevent EMT. Importantly, we discovered that CPX inhibited the development of kidney tumors in a xenograft mouse model. However, this study did not delve into the specific mechanism by which HMGA2 regulates the expression of Snail, a key transcription factor that plays an important role in the epithelial-mesenchymal transition (EMT) process. How HMGA2 regulates the expression of Snail still needs to be further explored in future studies. This will contribute to a more comprehensive understanding of the anti-tumor mechanism of CPX and provide a theoretical basis for the development of new therapeutic strategies. In addition, in future work, we intend to use ELISA (enzyme-linked immunosorbent assay) to confirm whether the secretion of M2-associated TGF-β cytokines is reduced after CPX treatment. This will further validate the role of CPX in inhibiting the TGF-β/Smads signaling pathway and provide more direct evidence for its anti-tumor mechanism. And we realized that adding immunohistochemistry (IHC) results could further strengthen the persuasiveness of the *in vivo* experiments. Therefore, we plan to include IHC experiments in future studies to more comprehensively validate the effects of CPX on HMGA2 and its downstream signaling pathways. Through IHC analysis, we will be able to more visually observe the expression and distribution of key proteins such as HMGA2, E-cadherin, and N-cadherin in tissue sections after CPX treatment. This will help to further understand the anti-tumor mechanism of CPX and provide a more solid theoretical basis for its clinical application.

HMGA2 induces an increase in the activity of the transcription factor E2F1 and promotes abnormal cell proliferation, thereby promoting the G2/M phase transition of the cell cycle and inducing tumor growth. HMGA2 increases the expression of CyclinD1 and CDK6, promoting the formation of CyclinD1 CDK6 complexes, accelerating the G1 phase of the cell cycle, promoting the G2/M phase transition, interfering with the cell cycle, and promoting tumorigenesis. The G1-phase process of the cell cycle, promotes the G2/M-phase transformation, interferes with cell cycle progression, and promotes tumorigenesis. Meanwhile, HMGA2 overexpression disrupted the DNA repair system. The cell cycle disorder and apoptosis inhibition of renal cancer are the key factors that make it difficult to completely cure renal cancer (Zhang et al., 2018; Kim et al., 2018). To enhance the occurrence, growth, and metastasis of renal cancer cells, HMGA2 can connect with Smad2 and Smad3 to form a complex that activates the expression of the target gene that encodes Snail, promotes the synthesis of Snail protein, and causes EMT in renal cancer cells. To further validate the effects of HMGA2 on the TGF-β/Smads and E2F1/Cyclin D1/CDK6 signaling pathways, we plan to conduct additional experiments by overexpressing or knocking down HMGA2 in future studies. This approach will provide a more comprehensive understanding of the specific mechanisms by which HMGA2 mediates the anti-tumor effects of CPX.

## 5 Conclusion

This study revealed the molecular mechanism leading to the occurrence and development of renal cell carcinoma and provides a new experimental and theoretical basis for CPX treatment of renal cell carcinoma. These findings support CPX as an anti-tumor agent targeting HMGA2 to prevent the development of kidney cancer. Despite our promising results, this study had several limitations. In this study, only a single renal carcinoma cell line, ACHN, was investigated, and further exploration is needed for cell lines such as renal carcinoma 786-O, 769-P, and others. We used two doses (10 and 30 mg/kg) of CPX to investigate whether it has a dose-dependent effect on the treatment of ACHN kidney cancer in mice. The results showed that 30 mg/kg CPX had a strong therapeutic effect on kidney cancer without significant hepatotoxicity. In terms of clinical translation, we will consider changing the frequency or method of administration at a later stage to avoid possible toxicities in patients with long-term treatment.

## Data Availability

The raw data supporting the conclusions of this article will be made available by the authors, without undue reservation.
